# Enter the Matrix: Factorization Uncovers Knowledge from Omics

**DOI:** 10.1016/j.tig.2018.07.003

**Published:** 2018-08-22

**Authors:** Genevieve L. Stein-O’Brien, Raman Arora, Aedin C. Culhane, Alexander V. Favorov, Lana X. Garmire, Casey S. Greene, Loyal A. Goff, Yifeng Li, Aloune Ngom, Michael F. Ochs, Yanxun Xu, Elana J. Fertig

**Affiliations:** 1 Department of Oncology, Division of Biostatistics and Bioinformatics, Sidney Kimmel Comprehensive Cancer Center, Johns Hopkins School of Medicine, Baltimore, MD, USA; 2 Department of Neuroscience, Johns Hopkins School of Medicine, Baltimore, MD, USA; 3 McKusick-Nathans Institute of Genetic Medicine, Johns Hopkins School of Medicine, Baltimore, MD, USA; 4 Department of Computer Science, Institute for Data Intensive Engineering and Science, Johns Hopkins University, Baltimore, MD, USA; 5 Department of Biostatistics and Computational Biology, Dana-Farber Cancer Institute, Boston, MA, USA; 6 Department of Biostatistics, Harvard TH Chan School of Public Health, Boston, MA, USA; 7 Vavilov Institute of General Genetics, Moscow, Russia; 8 University of Hawaii Cancer Center, Honolulu, HI, USA; 9 Department of Systems Pharmacology and Translational Therapeutics, Perelman School of Medicine, University of Pennsylvania, PA, USA; 10 Childhood Cancer Data Lab, Alex’s Lemonade Stand Foundation, PA,USA; 11 Digital Technologies Research Centre, National Research Council of Canada, Ottawa, ON, Canada; 12 School of Computer Science, University of Windsor, Windsor, ON, Canada; 13 Department of Mathematics and Statistics, The College of New Jersey, Ewing, NJ, USA; 14 Department of Applied Mathematics and Statistics, Whiting School of Engineering, Johns Hopkins University, Baltimore, MD, USA

## Abstract

Omics data contain signals from the molecular, physical, and kinetic inter- and intracellular interactions that control biological systems. Matrix factorization (MF) techniquescan reveal low-dimensional structure from high-dimensional data that reflect these interactions. These techniques can uncover new biological knowledge from diverse high-throughput omics data in applications ranging from pathway discovery to timecourse analysis. We review exemplary applications of MF for systems-level analyses. We discuss appropriate applications of these methods, their limitations, and focus on the analysis of results to facilitate optimal biological interpretation. The inference of biologically relevant features with MF enables discovery from high-throughput data beyond the limits of current biological knowledge - answering questions from high-dimensional data that we have not yet thought to ask.

## Determining the Dimensions of Biology from Omics Data

**Figure 1.**
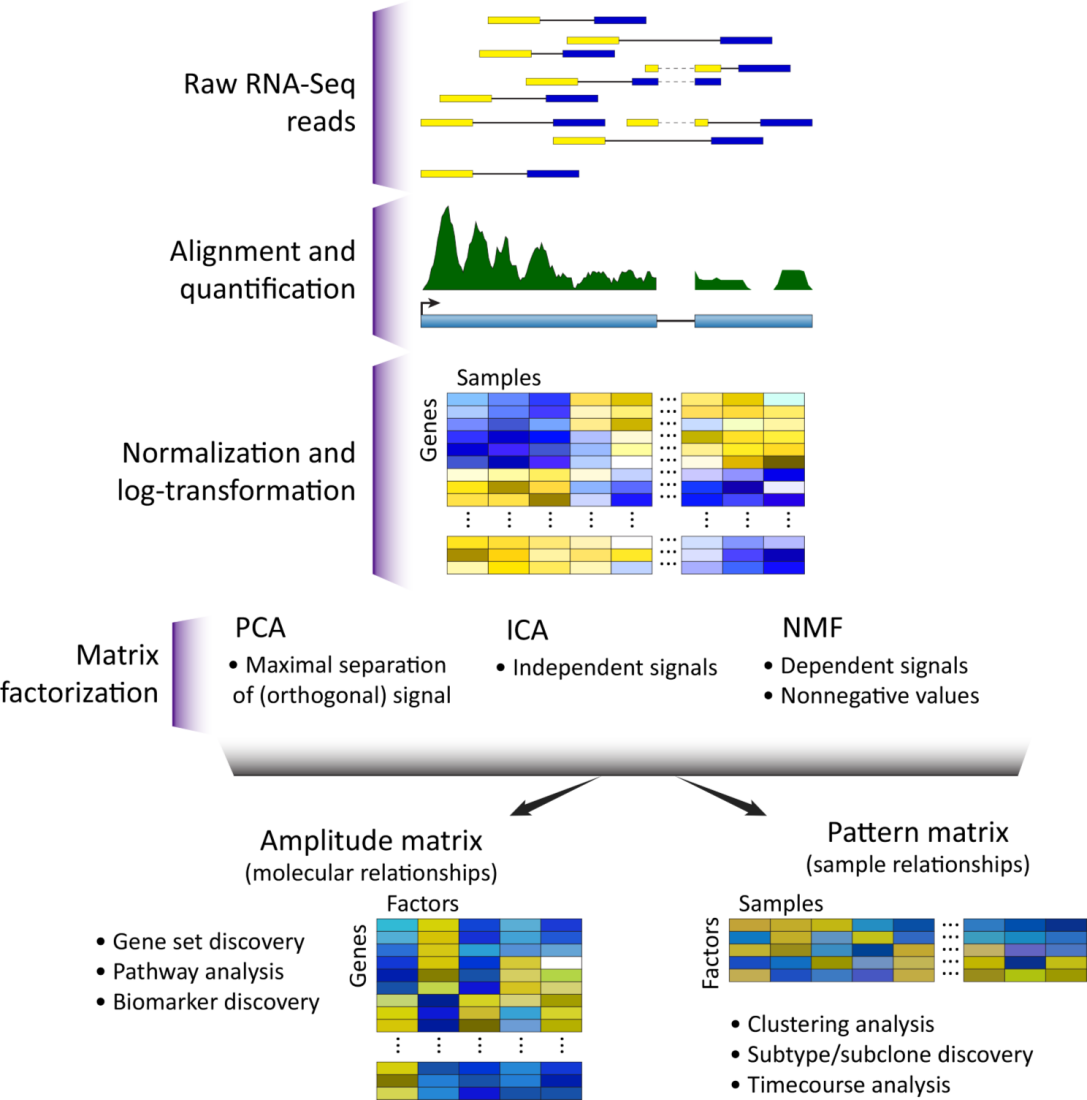
Omics Technologies Yield a Data Matrix That Can Be Interpreted through MF. The data matrixfrom omics has each sample as a column and each observed molecular value (expression counts, methylation Levels, protein concentrations, etc.) as a row. This data matrix is preprocessed with techniques specific to each measurement technology, and is then input to a matrix factorization (MF) technique for analysis. MF decomposes the preprocessed data matrix into two related matrices that represent its sources of variation: an amplitude matrix and a pattern matrix. The rows of the amplitude matrix quantify the sources of variation among the molecular observations, and the columns of the pattern matrix quantify the sources of variation among the samples. Abbreviations: ICA, independent component analysis; NMF, non-negative matrix factorization; PCA, principal component analysis.

High-throughput technologies have ushered in an era of big data in biology [1,2] and empowered *in silico* experimentation which is poised to characterize **complex biological processes** (CBPs; see Glossary) [3]. The natural representation of high-dimensional biological data is a matrix of the measured values (expression counts, methylation levels, protein concentrations, etc.) in rows and individual samples in columns (Figure 1). Columns corresponding to experimental replicates, or samples with similar phenotypes, will have values from the same distribution of biological variation. Related structures in the data are observed because they share one or more CBP. The activity of CBPs need not be identical in each sample. In these cases, the values of all molecular components that are associated with a CBP will change proportionally to the relative activity of that CBP. These phenotypes and CBP activities are often unknown *a priori,* requiring computational techniques for **unsupervised learning** to discover CBPs directly from the biological data.

The relationships between CBPs and similarities between samples constrain high-dimensional datasets to have low-dimensional structure. The number of genes, proteins, and pathways that are concurrently active within any cell is constrained by its energy and free-molecule limitations [4]. Only a characteristic subset of CBPs will be active in any cell at a given time. Thus, for a dataset where columns share CBP, a low-dimensional structure can be extracted which is smaller than either the number of rows or the number of columns.

**Matrix factorization** (MF) is a class of unsupervised techniques that provide a set of principled approaches to parsimoniously reveal the low-dimensional structure while preserving as much information as possible from the original data. MF is also referred to as matrix decomposition,and the corresponding inference problem as deconvolution. Other reviews discuss the mathematical and technical details of MF techniques [5–8] and their applications to microarray data [9]. We focus here on the biological applications of MF techniques and the interpretation of their results since the advent of sequencing technologies. We describe a variety of MF techniques applied to high-throughput data analysis, and compare and contrast their use for biological inference from bulk and single-cell data.

## Workflow for MF Analysis

After data preprocessing, most high-throughput molecular datasets can be represented as a matrix in which each element contains the measurement of a single molecule in a single experimental condition. In the example of RNA sequencing (RNA-seq), the number of short reads from each gene are summarized into gene level counts. The resulting high-dimensional dataset is formulated by representing these gene level counts for each sample as a column in the data matrix (Figure 1). MF methods can then be applied to these count matrices to learn CBPs from the data. Many MF techniques described are for RNA-seq data that are preprocessed using log transformation [10] or models of sequencing depth [11], while others directly model read counts [12].

Most examples featured in this review are based on such preprocessed RNA-seq analysis of gene counts or log-transformed gene counts. We note that applications of MF are not limited to this data modality or this preprocessing pipeline. For example, MF has also been applied to define mutational signatures in cancer [13,14], allele combinations in phenotypes [15], transcript regulation of genes [16], and distributions of transcript lengths [17], as well as to discriminate peptides in mass spectrometry proteomics [18]. To apply MF to other data modalities, they must also be properly preprocessed into a data matrix with a distribution appropriate to the MF analysis method.

**Figure 2.**
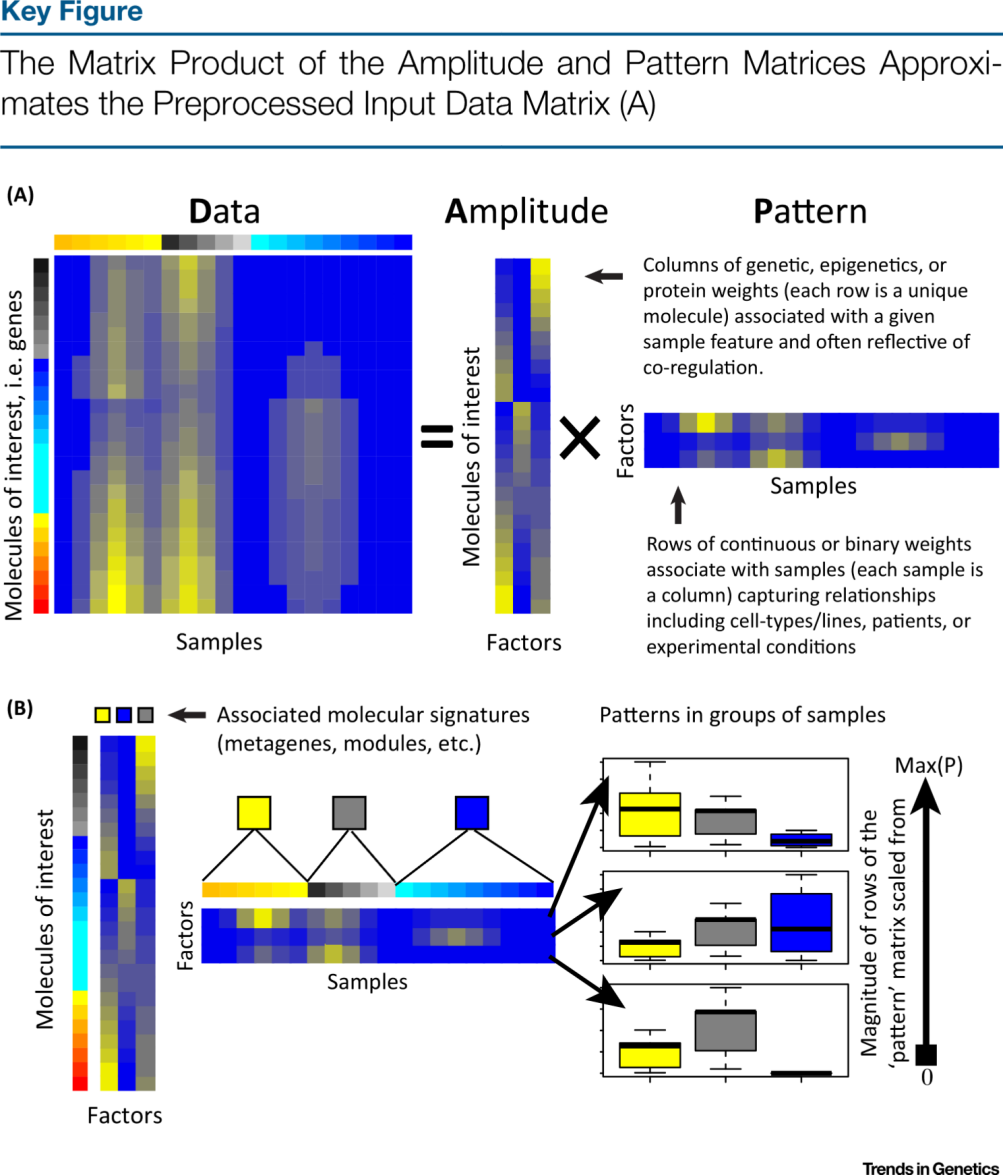
The number of columns of the amplitude matrix equals the number of rows in the pattern matrix, and represents the number of dimensions in the low-dimensional representation of the data. Ideally, a pair of one column in the amplitude matrix and the corresponding row of the pattern matrix represents a distinct source of biological, experimental, and technical variation in each sample (called complex biological processes, CBPs). (B) The values in the column of the amplitude matrix then represent the relative weights of each molecule in the CBP, and the values in the row of the pattern matrix represent its relative role in each sample. Plotting of the values of each pattern for a pre-determined sample grouping (here indicated by yellow, grey, and blue) in a boxplot as an example of a visualization technique for the pattern matrix. Abbreviation: Max(P), maximum value of each row of the pattern matrix.

When applied to high-throughput omics data, MF techniques learn two matrices: one describes the structure between features (e.g., genes) and another describes the structure between samples (Figure 2, Key Figure). We call the former feature-level matrix the **amplitude matrix** and the latter sample-level matrix the **pattern matrix**. Additional terms have been coined for the amplitude and pattern matrices based upon the MF problem applied and on the specific application to high-throughput biological data (Box 1). The values in each column of the amplitude matrix are continuous weights describing the relative contribution of a molecule in each inferred factor. In cases where factors distinguish between CBPs, the relative weights of these molecules can be associated with functional pathways. The same molecule may have high values in multiple columns of the amplitude matrix. Thus, MF techniques are able to account for the cumulative effect of genes that participate in multiple pathways.

Whereas each column of the amplitude matrix describes the relative contributions of molecules to a factor, each row of the pattern matrix describes the relative contributions of samples to a factor (Figure 2). Sample groups can be learned by comparing the relative weights in each row of the pattern matrix. The pattern matrix from MF can also be binarized to perform clustering [19,20] or kept as continuous values to define relationships between samples [21–23]. In the same way as molecules with high weights within a column of the amplitude matrix are associated with a common pathway, samples with high weights within a row of the pattern matrix can be assumed to share a common phenotype or CBP.

The optimal number of columns of the amplitude matrix and rows of the pattern matrix is often referred to as the dimensionality of the MF problem, and learning this value remains an open problem for the MF research community [24,25]. We also note that MF is not a single computational method. Instead, there is a wide body of literature on numerous MF techniques that have been applied throughout computational biology. The properties of both the amplitude and pattern matrices, and subsequently the interpretation of their values, depend crucially on the specific MF problem and the algorithm selected for analysis.

## MF Techniques: PCA, ICA, and NMF

There are numerous approaches to MF. The three most prominent MF approaches are **principal component analysis** (PCA), **independent component analysis** (ICA), and **non-negative matrix factorization** (NMF). Each of these techniques has a distinct mathematical formulation of a distinct MF problem (as described in the supplemental information online and in other reviews [5,8,26–29]).

Briefly, PCA finds dominant sources of variation in high-dimensional datasets, inferring genes that distinguish between samples. Maximizing the variability captured in specific factors, as opposed to spreading relatively evenly among factors, may mix the signal from multiple CBPs in a single component. Therefore, PCA may conflate processes that sometimes occur and complicate interpretation of the amplitude matrix for defining data-driven gene sets or the inference of specific CBPs.

ICA and NMF learn distinct processes from an input data matrix using different techniques. ICA learns factors that are statistically independent, resulting in more accurate association with literature-derived gene sets [30–32]. NMF methods constrain all elements of the amplitude and pattern matrices to be greater than or equal to zero [33,34]. Whereas the features in PCA can be ranked by the extent to which they explain the variation in the data, the features in both ICA and NMF are assumed to have equal weight. NMF is well suited to transcriptional data, which is typically non-negative itself, and semi-NMF is also applicable to data that can have negative values. The assumptions of NMF model both the additive nature of CBPs and parsimony, generating solutions that are biologically intuitive to interpret [35].

The solutions from both ICA and NMF may vary depending upon the initialization of the algorithm, leading to disparate amplitude and pattern matrices. Therefore, it is crucial to ensure that particular solution used for analysis provides an optimal and robust solution before using the results of the factorization to interpret CBPs. We previously found that Bayesian techniques to solve NMF have more robust amplitude matrices than gradient-based techniques, and thus generate more accurate associations between the values in the amplitude matrix and functional pathways [5,36]. Other studies have found that gradient-based approaches have similar computational performance to their Bayesian counterparts [37], and new techniques are being developed to enhance the stability of the factorization [38]. Further integrated computational/ experimental investigation is necessary to assess the biological relevance of solutions from both classes of techniques and their robustness. These associations also depend crucially on the input data. Therefore, to learn CBPs from data, MF must be applied to datasets with sufficient measurements of the experimental perturbations or conditions relevant to the specific biological problem being addressed.

## Sample Application to Genotype-Tissue Expression (GTEx) Project Gene Expression Data of Postmortem Tissues

**Figure 3.**
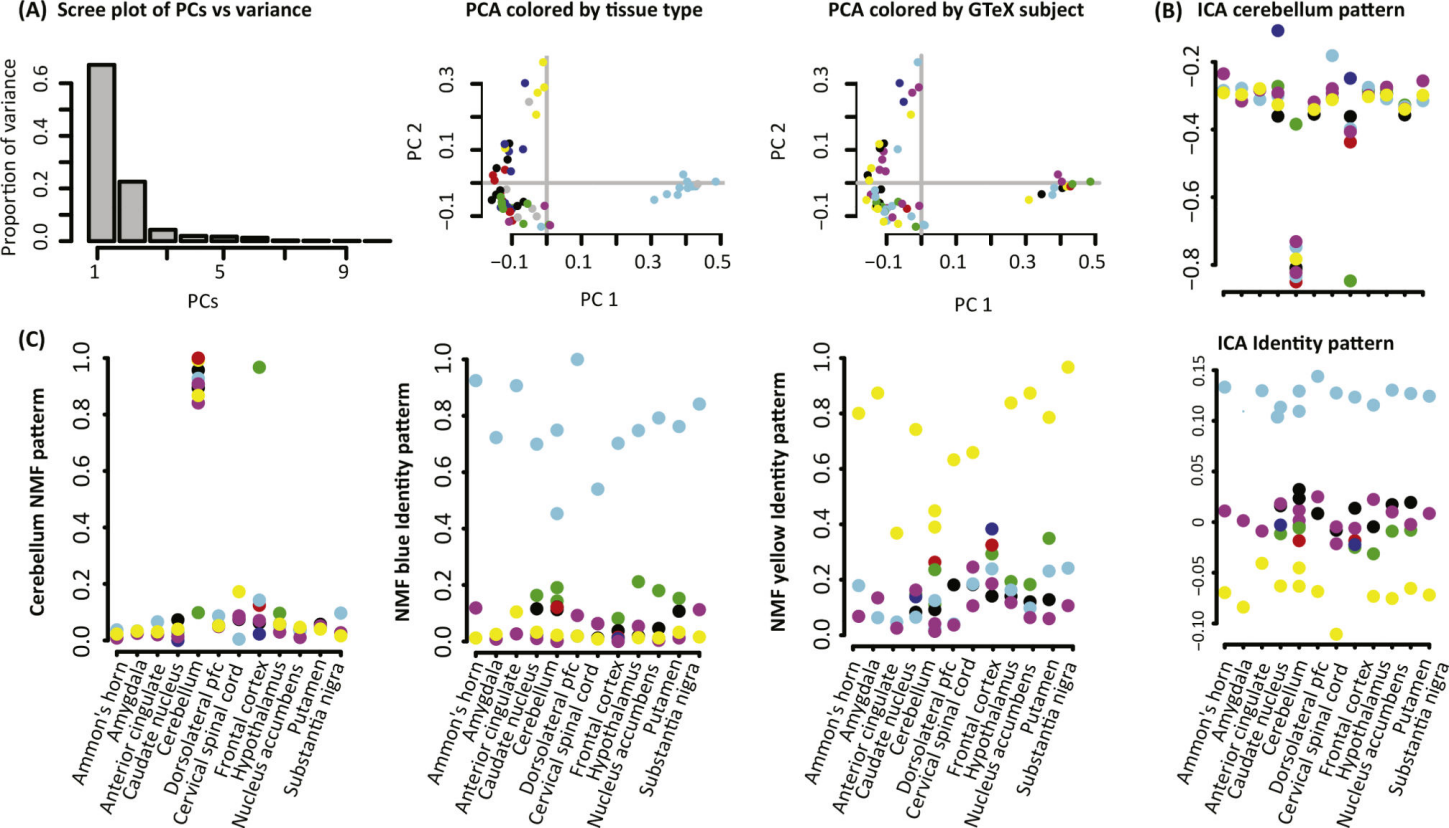
Comparison of Pattern Matrix From Matrix Factorization (MF) in Postmortem Tissue Samples from GTEx. (A) PCA finds factors in rows of the pattern matrix that can be ranked by the amount of variation that they explain in the data, as illustrated in a scree plot. PCA analyses typically plot the first two principal components (PCs; rows of the pattern matrix) to assess sample clustering. Points are colored by tissue type annotations from GTEx (left), where Ammon’s horn refers to the hippocampus, and donor (right). In GTEx data, the cerebellum (light blue) and first cervical spinal cord (yellow) cluster separately from all other brain tissues, but no separation between individuals is observed. (B) ICA finds factors associated with independent sources of variation, and therefore cannot be ranked in a scree plot. The relative absolute value of the magnitude of each element in the pattern matrix indicates the extent to which that sample contributes to the corresponding source of variation. The sign of the values indicate over- or underexpression in that factor depending on the sign of the corresponding gene weights in the amplitude matrix. As a result, the values can be plotted on the y axis against known covariates on the x axis to directly interpret the relationship between samples. When applied to GTEx, we observe one pattern associated with cerebellum, another pattern that has large positive values for one donor and large negative values for another donor, and eight other patterns associated with other sources ofvariation (supplemental information online). (C) NMF findsfactors that are both non-negative and not ranked by relative importance, similarly to ICA. The value of the pattern matrix indicates the extent to which each sample contributes to an inferred source of variation and is associated with overexpression of corresponding gene weights in the amplitude matrix. Values of the pattern matrix can be plotted similarly to ICA. When applied to GTEx, we observe one pattern associated with cerebellum, two more patterns associated with the two donors that were assigned to a single pattern in ICA, and seven other patterns associated with other sources of variation (supplemental information online). Abbreviations: GTEx, Genotype-Tissue Expression (GTEx) project; ICA, independent component analysis; NMF, non-negative matrix factorization; PCA, principal component analysis.

Applying multiple types of MF techniques to the same dataset or a single MF to distinct subsets of a dataset can also find distinct sources of variability. Selecting which MF method to use to learn relevant CBPs from a dataset is then crucial, and developing standardized metrics to choose between them is an active area of research in computational biology. To illustrate the differences between MF methods, we apply PCA, ICA (CRAN package fastICA [39]), and NMF (R/Bioconductor package CoGAPS [40,41]) to a single dataset from postmortem samples in the GTEx project [42] (Figure 3). Specifically, we select a subset of GTEx data containing 12 brain tissues in the GTEx data for seven individuals for which we had previously performed only NMF analysis [41] (codes are provided in the supplemental information online). We select this problem because the CBPs (tissue and individual) are readily separated and are known a *priori,* providing a known ground truth to facilitate comparison of methods.

First we apply PCA to this sample GTEx dataset (Figure 3A). The components learned from PCA can be ranked by the amount of variation that they explain in the data, with the first two components explaining 89.6% of the variation in this dataset. The amount of variation explained by these components can be used to determine the optimal dimensionality for PCA [24]. A typical PCA analysis explores the association between these components and biological covariates in low-dimensional plots in which each axis is defined by the weights in one row of the pattern matrix (Figure 3A). Similarly to clustering analyses, these plots can be used to determine which biological features can be separated from the data. In this application, we observe that the cerebellum (light blue) and first cervical spinal cord (yellow) cluster separately from all other brain tissues (PC1 and PC2, or rows 1 and 2 of the pattern matrix, respectively). No separation between individuals is observed in this PCA analysis.

In contrast to PCA, the components of ICA and NMF cannot be ranked by percent variation explained. Instead, each row of the pattern matrix is an equally important CBP in the data. Therefore, these patterns are plotted independently relative to biological covariates, and not relative to one another as in PCA analyses. When applied to the sample GTEx dataset, rows of the pattern matrix from both ICA (Figure 3B) and NMF (Figure 3C) also distinguish the cerebellum, similarly to PCA. Whereas PC1 has large positive values for the cerebellum and large negative values for the other brain tissues, both NMF and ICA have large absolute value only for the tissue of interest and are near zero for other tissues. As a result, both ICA and NMF provide tissue-specific patterns and PCA provides tissue-segregating patterns. We note that the magnitude of all these patterns is unit-less, reflecting the relative weights of each sample in the pattern and not a measurable quantity. Because gene weights can be either positive or negative in ICA, the patterns weights can also. By contrast, by construction the NMF values are all non-negative. As a result, the pattern corresponding to the cerebellum samples from ICA is still specific to that tissue, with genes overexpressed in this tissue having negative weights in the corresponding column of the amplitude matrix, and genes underexpressed in that tissue having positive weights. Indeed, the gene weights in the column of the amplitude matrix corresponding to the NMF cerebellum pattern are significantly anti-correlated with the gene weights in the column of the amplitude matrix corresponding to the ICA cerebellum pattern (*R* = –0.72, *P* <2 × 10^−16^).

In contrast to PCA, both ICA and NMF also infer patterns that distinguish individuals with common weights across all tissues. In the NMF analysis, each individual has a separate pattern. The ICA analysis has a single pattern that has large positive values for one of the individuals distinguished in one NMF pattern, and large negative values for another individual distinguished in different NMF pattern. This discrepancy highlights the difference between inferring independent sources of variation with ICA and NMF. Specifically, ICA may combine multiple CBPs using common genes whose sign changes by experimental condition, whereas NMF will find CBPs that are additive, corresponding only to overexpression of genes in that condition.

PCA, ICA, and NMF are equally valid, and their distinct formulation gives rise to the distinct features observed in the data. Applications of multiple types of MF techniques, or even the same MF algorithm with different parameters, may infer several CBPs or phenotypes within a single dataset, in essence providing answers to different questions. We further note that the specific techniques for PCA, ICA, and NMF selected for analysis in this example are only one of a multitude of variants of techniques which have been developed for computational biology. Applying multiple types of MF techniques to the same dataset, or a single MF to distinct subsets of a dataset, can also find distinct sources of variability. However, the general properties of these methods will remain and be consistent with our example. Briefly, we observe in this example that PCA finds sources of separation in the data, whereas both ICA and NMF find independent sources of variation. ICA can find both over- and underexpression of genes in a single CBP, whereas NMF can find only overexpressed genes in a single CBP. As a result, ICA may better model both repression and activation than NMF, but as a side effect may have greater mixture of CBPs than NMF.

Regardless of the technique selected, the results will also be sensitive to the input data. For example, a different NMF-class algorithm called ‘grade of membership’ (GOM) was also applied to a larger set of postmortem samples in GTEx. This algorithm found a pattern that combined all samples from brain regions when applied to all tissue samples in GTEx, but separated the distinct brain regions when applied only to tissue samples from the brain. Thus, applying multiple types of MF techniques to the same dataset, or a single MF to distinct subsets of a dataset, can also find distinct sources of variability that are essential for exploratory data analysis.

## Further Example Applications To Represent Cell Types, Disease Subtypes, Population Stratification, Tissue Composition, and Tumor Clonality with the Pattern Matrix

Exactly as we observed in the GTEx example, a single factorization of complex datasets can find multiple distinct sources of variation. For example, the power of MF to identify multiple sources of variation was seen when multiple technical factors from sample processing and biological factors were discovered in an ICA of gene expression profiles of 198 bladder cancer samples [43]. One factor in the pattern matrix of this analysis defined a CBP associated with gender. Because ICA simultaneously accounts for multiple factors in the data as separate rows in the same matrix, each row can fully distinguish a single biological grouping from the data.

Analysis of a single dataset with one MF algorithm using different numbers of factors can reflect a hierarchy of biological processes. For example, applying CoGAPS to data from a set of head and neck tumors and normal controls for a range of dimensionalities was able to separate tumor and normal samples when limited to two patterns, but further decomposed the tumor samples into the two dominant clinical subtypes of head and neck cancer when identifying five patterns for the same data [44]. The hierarchical relationship between patterns has been used to assess the robustness of patterns to quantify the optimality of the factorization [45] and learn the optimal dimensionality of the factorization [46]. Other algorithms use statistical metrics to estimate the number of factors [12,47]. While these algorithms quantify fit to the data, they may disregard the hierarchical nature of distinct CBPs learned by factoring biological data into multiple dimensions. This observation highlights the complexity of estimating the number of factors for optimal MF analysis of biological data (see Outstanding Questions).

Moreover, application of different MF algorithms to the same dataset can give different sample groupings that reflect biology. For example, in population genetics a grouping inferred from GWAS which distinguishes ancestry is equally valid to a grouping inferred from the same GWAS data which distinguishes disease risk. The application of PCA to SNP data from 3000 European individuals [48] demonstrates inference of sample corelationships using the pattern matrix, and found that much of the variation in DNA sequence is explained by the longitude and latitude of the country of origin of an individual. In addition, statistical models can be formulated assuming that the inheritance of an individual arises from proportions of ancestry in distinct populations through genetic admixture [48]. An MF-based technique called sparse factor analysis also distinguishes between these populations using GWAS data [49]. These analyses demonstrate that the ancestry of each individual is a dominant source of variation in DNA sequence. At the same time, sources of variation in GWAS data arise from variants that give rise to disease risk, which can be shared among individuals with diverse genetic backgrounds [50]. In the same way as we observed in our GTEx example, the application of multiple MF techniques is essential to determine each source of variation in GWAS studies.

Mixtures of cell types in biological samples introduce a further degree of complexity to MF analysis of biological variation in their molecular data. **Computational microdissection** algorithms estimate the proportion of distinct cell types within a bulk sample by applying MF to genes whose expression is uniquely associated with each cell type [51]. Subsetting the data to different genes may give rise to different factors that represent different CBPs. Nonetheless, CoGAPS NMF analysis of data subsets that were obtained by selecting equally sized sets of random genes found that the pattern matrices were consistent for each random geneset in the expression data [41,52]. These results suggest that the dependency of an MF on the specific genes used for analysis may depend on the heterogeneity of the signal in the data matrix.

Cellular and molecular heterogeneity poses a particular challenge to MF analysis in cancer genomics. Even a pure tumor tissue can contain numerous subclones owing to the accumulation of different driver events during tumor evolution. New MF techniques have been developed to estimate the proportion of the tumor that arises from each subclone [47,53–56]. Assumptions about the evolutionary mechanisms of the accumulation of molecular alterations can also be encoded in the factorization to model the resulting heterogeneity of these clones [12,47]. These studies demonstrate that encoding prior knowledge into MF can focus the resulting factors to reflect one of the equally valid biological groupings within the data.

## From Snapshots to Moving Pictures: Simplifying Timecourse Analysis

**Figure 4.**
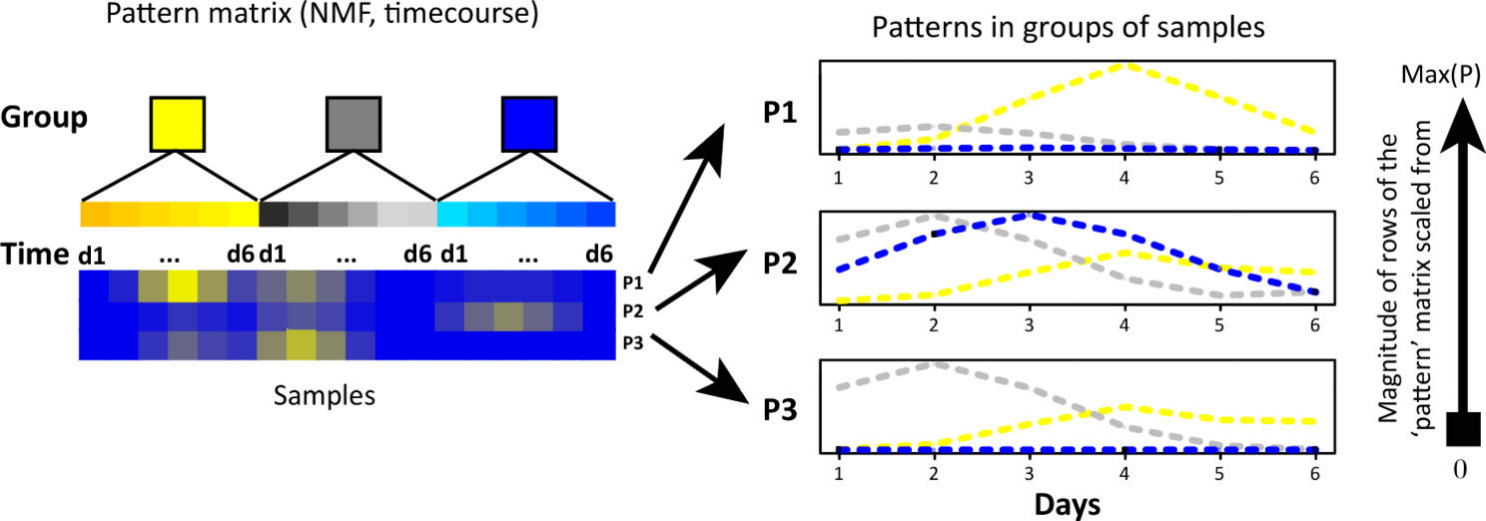
Samples Correspond to Timepoints; the Rows of the Pattern Matrix Can Be Plotted as a Function of Time and Sample Condition To Infer the Dynamics of Complex Biological Processes (CBPs). Abbreviations: d1-d6, days 1–6; max(P), maximum value of each row of the pattern matrix; NMF, non-negative matrix factorization; P1–3, patterns 1–3.

Entwined in the challenge of decomposing cell types and subpopulations is the fact that CBPs change over time. High-throughput timecourse datasets are emerging in the literature to account for the dynamics of biological systems. The central goal of timecourse analysis is to determine the extent to which molecules change over time in response to perturbations (e.g., developmental time, environmental factors, disease processes, or therapeutic treatments). Associating molecular alterations often relies on specialized bioinformatics techniques for timecourse analysis [57,58]. MF analyses can naturally infer changes in CBPs over time when applied to timecourse data because the continuous weights for each sample in the pattern matrix can vary among samples collected across distinct timepoints. The relative weights of rows of the pattern matrix can encode the timing of regulatory dynamics directly from the data (Figure 4). Nonetheless, most MF algorithms for timecourse analysis do not encode the known timepoints or retain their relative ordering. Methods that specifically use these temporal data are currently an active area of research.

Both ICA and NMF were found to have signatures characterizing the yeast cell cycle and metabolism in early timecourse microarray experiments [59,60]. The sparse NMF techniques using Bayesian methods had patterns that reflected the smooth dynamics of these phases [36,59]. This approach has been shown to simultaneously learn pathway inhibition and transitory responses to chemical perturbation of cancer cells [61] and relate the changes in phospho-proteomic trajectories between multiple therapies [62]. Similar analysis of healthy brain tissues learned the dynamics of transcriptional alterations that are common to the aging process in multiple individuals [52]. MF techniques designed for cancer subclones described in the previous section have also been applied to repeat samples to learn the dynamics of cancer development, thereby elucidating the molecular mechanisms that give rise to therapeutic resistance and metastasis. Even if there are the same number of biological features, the rate or timing of related features in different molecular modalities may be offset [63]. These discrepancies by data modality suggest that different regulatory mechanisms may be responsible for initiating and stabilizing the malignant phenotype [63].

## Data-Driven Gene Sets from MF Provide Context-Dependent Coregulated Gene Modules and Pathway Annotations

Genomic data are often interpreted by identifying molecular changes in sets of genes annotated to functionally related modules or pathways, called gene sets [64,65]. Often the associations between gene sets and functions are based upon manual curation of the literature [66,67]. Such set-level interpretations often lack important contextual information [64,68,69], and cannot describe genes of unknown function or genes associated with new functional mechanisms.

**Figure 5.**
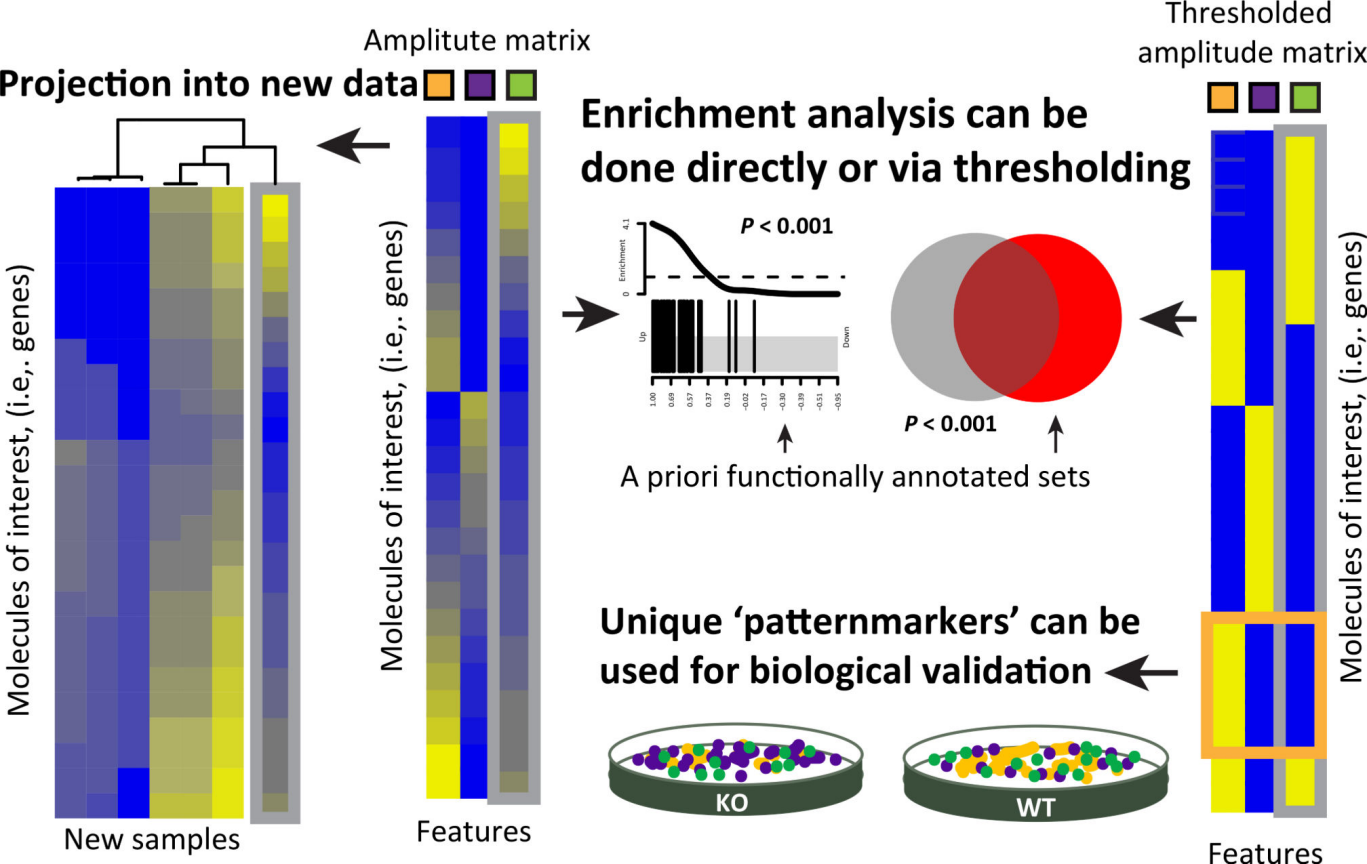
The Amplitude Matrix from Matrix Factorization (MF) Can Be Used to Derive Data-Driven Molecular Signatures Associated with a Complex Biological Process (CBP). The columns of the amplitude matrix contain continuous weights describing the relative contribution of a molecule to a CBP (center panel; indicated by the orange, purple, and green boxes). The resulting molecular signature can be analyzed in a new dataset to determine the samples in which each previously detected CBP occurs, and thereby assess function in a new experiment. This comparison may be done by comparing the continuous weights in each column of the amplitude matrix directly to the new dataset (left). The amplitude matrix may also be used in traditional gene-set analysis (right). Traditional gene-set analysis using literature curated gene sets can be performed on the values in each column of the amplitude matrix to identify whether a CBP is occurring in the input data. Data-driven gene sets can also be defined from this matrix directly using binarization, and used in place of literature-curated gene sets to query CBPs in a new dataset. Sets defined from molecules with high weights in the amplitude matrix comprise signatures akin to many curated gene-set resources, whereas molecules that are most uniquely associated with a specific factor (purple box) may be biomarkers. Abbreviations, KO, knockout; WT, wild type.

The amplitude matrix from MF analysis can be used both for literature-based gene-set analysis and to define new data-driven gene signatures (Figure 5). Standard gene-set analysis can be applied directly to the values in each column of the amplitude matrix to associate the inferred factors with literature-curated sets. New, context-dependent gene sets can also be learned from the values in the amplitude matrix. Gene-set annotations are often binary. Thresholding techniques to select which genes belong to a pathway from the amplitude matrix for binary membership provide an output similar to gene sets in databases [70,71]. Other studies also integrate the literature-derived gene signatures in these thresholds to refine the context of pathway databases [36,72]. The genes derived from these binarizations can be used as inputs to pathway analyses from differential expression statistics in independent datasets (Figure 5, right), and are analogous to the hierarchical clustering-based gene modules [73] and gene expression signatures from public domain studies in the MSigDB gene-set database [74]. Another means of binarizing the data is to find genes that are most uniquely associated with a specific pattern to use as biomarkers of the cell type or process associated with that pattern [41,75]. Selecting genes based upon these statistics can facilitate visualization of the CBPs in high-dimensional data [41]. Whereas binarization of genes with high weights can associate a single gene with multiple CBPs, the statistics for unique associations link a gene with only one CBP. Therefore, these statistics also define specific genes that may be biomarkers of the cell type/state or a process [41] (Figure 5, right).

Although binary pathway models are substantially easier to interpret, continuous values from the original factorization provide a better model of the input data. Weighted gene signatures have been shown to be more robust to noise and missing values in the data [76]. If the expression level of a gene is poorly measured in a sample, other genes in the same factor can imply the actual expression level of the gene in question. By considering each gene in the context of all other genes, factorization improves the robustness of the findings. Further, continuous signatures can be associated directly with other samples using projection methods [76,77] or profile correspondence methods [78].

## MF Enables Unbiased Exploration of Single-Cell Data for Phenotypes and Molecular Processes

MFapproaches are a natural choice in single-cell RNA-seq (scRNA-seq) data analysis owing to the high dimensionality of the data, and are used to identify and remove batch effects, summarize CBPs, and annotate cell types in the data [79–82]. Whereas MF analysis of bulk data dissects groups from a small subset of samples, the analysis in scRNA-seq data aggregates cells into groups of common cell types or CBPs [75,83]. Often these analyses are performed on a subset of the data containing the most variable genes. Newer computationally efficient methods are being developed to enable factorization of large omics datasets for genome-wide analysis [84]. Biological knowledge can be encoded with a class of MF algorithms that summarize factors using gene sets [79,85,86].

Most MF techniques developed for bulk omics data assume that the gene expression changes from CBPs are additive. This assumption is violated in scRNA-seq data. One reason for the violation of the additive assumption in MF is the inability to distinguish true zeros from missing values. Imputation methods for preprocessing [81,87] or newer MF algorithms that model missing data are essential for scRNA-seq data. Branching of trajectories of cellular states and lack of cell-cycle synchronization in scRNA-seq data further violate the additive assumption in MF. New nonlinear factorization techniques are being developed to enhance visualization of trajectory structures in single-cell data [80,88–90] in these cases. However, the results from these methods cannot be interpreted in the same way as those from MF algorithms. In particular, the low-dimensional solutions from these methods are not necessarily useable to reconstruct the original high-dimensional data.

## Concluding Remarks

MF encompasses a versatile class of techniques with broad applications to unsupervised clustering, biological pattern discovery, component identification, and prediction. Since MF was first applied to microarray data analysis in the early 2000s [59,91–93], the breadth of MF problems and algorithms for high-throughput biology has grown with their broad applications. MF problems are ubiquitous in the computational sciences, with examples including unsupervised feature learning [94–99], clustering and metric learning [100–102], latent Dirichlet allocation [103], subspace learning [104–109], multiview learning [110], matrix completion [111], multitask learning [112], semi-supervised learning [113], compressed sensing [114], and similarity-based learning [115,116]. Dimension reduction of biological data with MF highlights perspectives and questions that investigators have not yet considered, and also enables tractable exploration of otherwise massive datasets. As the size of these datasets grow, it is crucial to develop new algorithms to solve MF problems that scale with the ever-increasing size of omics data [37,41,117,118]. MF algorithms can also be extended for simultaneous analysis of data from multiple data modalities, enabling genomic data integration [7,8,119]. Techniques that extend this integrated MF frame-work, including Bayesian group factor analysis [8] and tensor decomposition [120–123], can also analyze datasets across different molecular levels [124]. Developing such data-integration techniques is an active area of research in both genomics and computational sciences.

Different classes of techniques solve MF, including gradient-based and probabilistic methods (supplemental information online). Distinct MF problems each aim to identify specific types of features. In some cases different algorithms will learn distinct features from the same dataset. Therefore, investigators may benefit from applying multiple techniques with different properties, or by carefully considering both the dataset and the question in selecting exactly the right technique for that question. Most such comparisons in the literature have been made by investigators who are developing MF methods. Unbiased assessments of the relative performance of different MF algorithms for different exploratory data analysis problems are essential to determine the relative strengths and weaknesses of each method for distinct biological problems. MF algorithms can be further tailored to the biological problem of interest using methods that also encode prior biological knowledge of the system underlying the measured dataset [125–127].

The features MF techniques extract are constrained by the dataset used to train them. These algorithms cannot learn unmeasured features, nor can they correct for complete overlap between technical artifacts and biological conditions. Thus, being mindful of experimental design when selecting datasets and choosing those that are broad enough to cover the relevant sources of variability is essential. Advances in MF and related techniques will be essential for powering systems-level analyses from big data (see Outstanding Questions).

## Supplementary Material

1

## Box 1. Common Terminology in the Literature

Historically, the independent discovery of MF in multiple fields including mathematics, computer science, and statistics created distinct terminologies that are often used interchangeably as analytical orthologs in genomics. For example, the term ‘factorization’ is often used interchangeablywith ‘decomposition’. Other terms, such as ‘features’, ‘components’, ‘latent variable’, or ‘latent factors’ are used to refer to relationships between either molecular measurements or samples, depending on the context.

The specific terminology for the amplitude and pattern matrices also varies according to the method, and are preferably labeled with different variable names. In PCA, the amplitude matrix is often called the score or rotation matrix, and pattern matrix called the loadings. In ICA, the amplitude matrix is called the unmixing matrix and the pattern matrix is called the source matrix. In NMF, the amplitude matrix is commonly called the weights matrix and the pattern matrix is called the features matrix.

Throughout this paper we use ‘amplitude matrix’ to refer to the matrix that contains vectors which represent relationships between molecular measurements. Other terms used in the literature for these molecular relationships include modules, meta-pathways, and signatures. Similarly, we use ‘pattern matrix’ to refer to the matrix that contains vectors which represent relationships between samples. Other literature terms for these sample-level relationships include patterns, metagenes, eigengenes, sources, and controlling factors.

## Outstanding Questions

How can the optimal number of factors be quantified? Increasing the number of factors in MF improves its approximation, but may cause overfitting. Computational metrics that balance these properties can be used to estimate the number of factors. Biologically driven metrics are also essential to assess whether this number represents the number of CBPs.

How can different factorizations be compared? Different MF algorithms identify different properties and CBPs from a single dataset. New techniques will be necessary to compare and merge the disparate but equally valid factorizations.

What techniques are best for efficient and optimal factorization? Large omics data-sets are common, particularly with single-cell technologies. Fast computational approaches to MF are required for the analysis of these data. These algorithms also require computational criteria to ensure that the solutions are robust.

How can fully nonlinear factorizations be performed on omics-scale data? Nonlinear extensions to MF have notable applications to single-cell data. Researchers often use low-dimensional representations of omics data learned from linear MF as inputs to nonlinear MF to account for both computational efficiency and the underlying mathematical assumptions of these nonlinear methods. Breakthroughs in theories for techniques such as kernel MF, deep MF, and manifold learning will enable fully nonlinear factorizations for large-scale single-cell data.

How can both gene regulatory relationships and distinct sources of technical variation be encoded in integrative analysis of data from different measurement technologies? Different measurement technologies yield data that follow unique distributions. CBPs and their timing also vary between types of measures. Integrative techniques must account for both the technical and biological sources of variation between disparate data modalities, including sparse or missing data. Such techniques are crucial to learning the regulatory relationships that drive CBPs and to modeling the multiscale nature of biological systems from omics data.

## Highlights

MFs techniques infer low-dimensional structure from high-dimensional omics data to enable visualization and inference of complex biological processes (CBPs).

Different MFs applied to the same data will learn different factors. Exploratory data analysis should employ multiple MFs, whereas a specific biological question should employ a specific MF tailored to that problem.

MFs learn two sets of low-dimensional representations (in each matrix factor) from high-dimensional data: one defining molecular relationships (amplitude) and another defining sample-level relationships (pattern).

Data-driven functional pathways, biomarkers, and epistatic interactions can be learned from the amplitude matrix.

Clustering, subtype discovery, *in silico* microdissection, and timecourse analysis are all enabled by analysis of the pattern matrix.

MF enables both multi-omics analyses and analyses of single-cell data.

## Glossary

Amplitude matrix: the matrix learned from MF that contains molecules in rows and factors in columns. Each column represents the relative contributions of the genes to a factor, and these can be used to define a molecular signature for a CBP.
Complex biological process (CBP): the coregulation or coordinated effect of multiple molecular species resulting in one or more phenotypes. Examples can range from activation of multiple proteins in a single cellular signaling pathway to epistatic regulation of development.
Computational microdissection: a computational method to learn the composition of a heterogeneous sample, for example the cell types in a tissue sample.
Independent component analysis (ICA): an MF technique that learns statistically independent factors.
Matrix factorization (MF): a technique to approximate a data matrix by the product of two matrices (Box 1), one of which we call the amplitude matrix and the other the pattern matrix.
Non-negative matrix factorization (NMF): an MF technique for which all elements of the amplitude and pattern matrices are greater than or equal to zero.
Pattern matrix: the matrix learned from MF that contains factors in rows and samples in columns. Each row represents the relative contributions of the samples to a factor, and these can be used to define the relative activity of CBPs in each sample.
Principal component analysis (PCA): an MF technique that learns orthogonal factors ordered by the relative amount of variation of the data that they explain.
Unsupervised learning: computational techniques to discover features from high-dimensional data, without reliance on prior knowledge of low-dimensional covariates.
