# Supplementary material for: Enter the Matrix: Factorization Uncovers Knowledge from Omics
Source: Trends Genet. Author manuscript; Available in PMC 2019 Oct 1. (PMC6309559; doi:10.1016/j.tig.2018.07.003)
Supplement: 1 [file NIHMS1510756-supplement-1.docx]

Enter the matrix: factorization uncovers knowledge from omics

Genevieve L. Stein-O’Brien et al.

# Mathematical description of matrix factorization

## Technical description of matrix factorization

Matrix factorization (MF) approximates an input matrix $X$, as a product of two matrices, $U$ and $V$, also referred to as factors.

In genomics, the input matrix $X$ typically represents a preprocessed data matrix that has $n$ molecular measurements as rows and $p$ biological samples as columns (i.e., a matrix of size $n\times p$). Often, not all of the row and column vectors of the data are equally informative. Exploratory data analysis aims to find a set of $k$ most informative dimensions or features, where $k$ is smaller than either $n$ or $p$. A $k$-dimensional representation that captures most of the “information” (say, for example, most of the variation) contained in the original data can then be used in its place in subsequent analysis.

MF solves this problem by approximating $X$ as a product of two “factor” matrices: $X\approx UV$, where the factor $U$ is of size $n\times k$ and the factor $V$ is of size $k\times p$. Here, we refer to $U$ as the amplitude matrix and $V$ as the pattern matrix. We note that alternative variable names to and and terminology are often used in the literature, depending on the application.

Finding matrices $U$ and $V$ requires a quantitative definition of how well the product $UV$ approximates $X$. One method for providing this definition is to use a loss function that quantifies the discrepancy between the approximation and the data, such as the mean square error and median absolute error. A common approach to solve for $U$ and $V$ is to apply an iterative approach such as a gradient-based method to minimize a loss function. Bayesian approaches to MF are alternatives to approximate the probabilistic relationship between $UV$ and $X$. Additional conditions can also be incorporated in both gradient-based and Bayesian MF to learn useful features, such as sparsity constraints to limit the number of non-zero matrix elements.

Both gradient-based and Bayesian MF approaches have numerous applications beyond genomics. These techniques are ubiquitous in unsupervised feature learning for big data analysis.

## Description of PCA, ICA, and NMF

MF can start with the same data matrix $X$ and then solve different problems to learn amplitude ($U$) and pattern ($V$) matrices with specific properties. An MF method requires: 1) the desired dimension $k$; 2) a loss function that measures the approximation $X\approx UV$; and 3) constraints on $U$ and/or $V$ that enforce a desired structure on the low-dimensional representation. Changes to any of these give rises to different MF problems with different low-dimensional representations of the data. Three common MF approaches are applied to genomics: PCA, ICA, and NMF.

PCA finds a low-dimensional representation of the data that maximizes the variation contained in the original data. In PCA, the columns of $U$ are orthogonal to each other describing non-overlapping structure in the data. Each column of represents the weight or loading of the data vector in the corresponding column of matrix $X$. In PCA, the columns of $U$ and rows of $V$ are ranked by the relative amount of variance they explain in the data. Thus, the first column of and row of explain most of the variation across molecular measurements and samples, respectively. Together, the $k$ vectors learned with PCA maximize the total variance in the data captured using any rank $k$ factorization. Geometrically, the vectors can be thought of as a set of orthogonal coordinate axes in high dimensional space, which represent the directions of maximal variation in the data.

ICA assumes that there are a set of $k$ independent sources of variation that give rise to the observed data matrix $X$. This method enforces that the columns of $U$ yields components that are statistically independent of each other. ICA is solved by minimizing the total mutual information between the $k$ estimated components. The resulting factors ideally represent independent sources of variation in the biological system. In ICA, often the $U$ matrix is often represented by the variable $A$ and the pattern matrix $S$. We note that ICA lacks identifiability for Gaussian data, which may limit applications of this method in genomics.

Non-negative matrix factorization (NMF) is a group of algorithms that constrains all elements of the $U$ and $V$ matrices to be greater than or equal to zero. The non-negativity constraint makes the representation purely additive, with no sources that can explain the data by removing signal. This non-negativity often results in NMF producing a sparse representation of the data. The additivity and sparsity make the $k$ features inferred from NMF easy to interpret as the set of active components of the data that will give the original data when added together. Often, $U$ is represented as the variable $W$ and $V$ by the variable $H$.

# GTEx example

## R packages and session info

library('fastICA')
library('pca3d')
sessionInfo()

## R version 3.3.3 (2017-03-06)
## Platform: x86_64-apple-darwin13.4.0 (64-bit)
## Running under: macOS Sierra 10.12.6
##
## locale:
## [1] en_US.UTF-8/en_US.UTF-8/en_US.UTF-8/C/en_US.UTF-8/en_US.UTF-8
##
## attached base packages:
## [1] stats graphics grDevices utils datasets methods base
##
## other attached packages:
## [1] pca3d_0.10 fastICA_1.2-1
##
## loaded via a namespace (and not attached):
## [1] rgl_0.98.1 Rcpp_0.12.14 digest_0.6.13 rprojroot_1.2
## [5] mime_0.5 R6_2.2.2 xtable_1.8-2 jsonlite_1.5
## [9] backports_1.1.0 magrittr_1.5 ellipse_0.3-8 evaluate_0.10.1
## [13] stringi_1.1.5 rmarkdown_1.5 tools_3.3.3 stringr_1.2.0
## [17] htmlwidgets_0.9 shiny_1.0.5 httpuv_1.3.5 yaml_2.1.16
## [21] htmltools_0.3.6 knitr_1.18

## Loading in GTEx data

load('GTExGeneRPKM.SUB10Brain.RData')

We load in the RPKM values from the subset of GTEx data in brain tissues which we previously analyzed with GWCoGAPS in Stein-O'Brien et al, *Bioinformatics*, 2017, doi: 10.1093/bioinformatics/btx058.

The tissue samples in this dataset are as follows:

length(unique(pd.GTExGeneRPKM.SUB10Brain$SMUBRTRM))

## [1] 12

unique(pd.GTExGeneRPKM.SUB10Brain$SMUBRTRM)

## [1] "amygdala"
## [2] "anterior cingulate cortex"
## [3] "caudate nucleus"
## [4] "cerebellum"
## [5] "frontal cortex"
## [6] "dorsolateral prefrontal cortex"
## [7] "Ammon's horn"
## [8] "hypothalamus"
## [9] "nucleus accumbens"
## [10] "putamen"
## [11] "first cervical spinal cord segment"
## [12] "substantia nigra"

The individuals in this dataset are as follows:

length(unique(pd.GTExGeneRPKM.SUB10Brain$SubjectID))

## [1] 7

table(pd.GTExGeneRPKM.SUB10Brain$SubjectID,
 pd.GTExGeneRPKM.SUB10Brain$SMUBRTRM)

##
## Ammon's horn amygdala anterior cingulate cortex
## GTEX-11GSP 0 0 0
## GTEX-11O72 0 0 0
## GTEX-11OF3 0 0 0
## GTEX-11TUW 0 0 0
## GTEX-Q2AG 1 1 1
## GTEX-QDT8 1 1 1
## GTEX-ZAB4 1 1 1
##
## caudate nucleus cerebellum dorsolateral prefrontal cortex
## GTEX-11GSP 1 2 1
## GTEX-11O72 0 1 0
## GTEX-11OF3 1 2 0
## GTEX-11TUW 1 0 0
## GTEX-Q2AG 1 2 1
## GTEX-QDT8 1 2 1
## GTEX-ZAB4 1 2 1
##
## first cervical spinal cord segment frontal cortex
## GTEX-11GSP 1 1
## GTEX-11O72 0 1
## GTEX-11OF3 0 1
## GTEX-11TUW 0 1
## GTEX-Q2AG 1 1
## GTEX-QDT8 2 1
## GTEX-ZAB4 1 0
##
## hypothalamus nucleus accumbens putamen substantia nigra
## GTEX-11GSP 1 1 1 0
## GTEX-11O72 0 0 0 0
## GTEX-11OF3 1 1 1 0
## GTEX-11TUW 0 0 0 0
## GTEX-Q2AG 1 1 1 1
## GTEX-QDT8 1 1 1 1
## GTEX-ZAB4 1 1 1 1

## CoGAPS (NMF) analysis from Stein-O'Brien et al, Bioinformatics, 2017

load('AP.fixed.SUB10Brain.nP10.nS15.RData')

par(mfrow=c(2,5))
for (i in 1:10) {
 plot(as.numeric(as.factor(pd.GTExGeneRPKM.SUB10Brain$SMUBRTRM)),AP.fixed$P[i,],
 col=as.numeric(as.factor(pd.GTExGeneRPKM.SUB10Brain$SubjectID)),axes=F,pch=19)
 axis(2)
 axis(1,at=1:length(levels(as.factor(pd.GTExGeneRPKM.SUB10Brain$SMUBRTRM))),
 labels=levels(as.factor(pd.GTExGeneRPKM.SUB10Brain$SMUBRTRM)),las=2)
}

## PCA analysis of GTEx data subset

dana <- p.GTExGeneRPKM.SUB10Brain.log2[row.names(AP.fixed$A),]

eigs <- princomp(cor(dana))$sdev^2
eigs <- eigs/sum(eigs)

barplot(eigs[1:10], ylab='Proportion of variance')

sum(eigs[1:2])

## [1] 0.8960462

par(mfrow=c(1,2))
pca2d(princomp(cor(dana)),col=as.numeric(as.factor(pd.GTExGeneRPKM.SUB10Brain$SMUBRTRM)))
title('PCA colored by tissue type')

pca2d(princomp(cor(dana)),col=as.numeric(as.factor(pd.GTExGeneRPKM.SUB10Brain$SubjectID)))
title('PCA colored by GTeX subject')

par(mfrow=c(1,2))
plot(c(1,0),c(1,0),col='white')
legend('topright',pch=19,col=1:length(levels(as.factor(pd.GTExGeneRPKM.SUB10Brain$SMUBRTRM))),
 legend = levels(as.factor(pd.GTExGeneRPKM.SUB10Brain$SMUBRTRM)),
 cex = 0.5)

plot(c(1,0),c(1,0),col='white')
legend('topright',pch=19,col=1:length(levels(as.factor(pd.GTExGeneRPKM.SUB10Brain$SubjectID))),
 legend = levels(as.factor(pd.GTExGeneRPKM.SUB10Brain$SubjectID)))

## ICA analysis of GTEx data subset

dana <- dana[apply(dana,1,sd)>0,]

set.seed(1234567)
ica <- list()
for (p in 10) {
 ica[[paste('p',p)]] <- fastICA(dana,n.comp = p)
}

par(mfrow=c(2,5))
for (i in 1:10) {
 plot(as.numeric(as.factor(pd.GTExGeneRPKM.SUB10Brain$SMUBRTRM)),ica$`p 10`$A[i,],
 col=as.numeric(as.factor(pd.GTExGeneRPKM.SUB10Brain$SubjectID)),axes=F,pch=19)
 axis(2)
 axis(1,at=1:length(levels(as.factor(pd.GTExGeneRPKM.SUB10Brain$SMUBRTRM))),
 labels=levels(as.factor(pd.GTExGeneRPKM.SUB10Brain$SMUBRTRM)),las=2)
}

## Comparison of CoGAPS NMF and ICA cerebellum patterns

cor.test(ica[['p 10']]$S[,8],AP.fixed$A[row.names(dana),3])

##
## Pearson's product-moment correlation
##
## data: ica[["p 10"]]$S[, 8] and AP.fixed$A[row.names(dana), 3]
## t = -226.13, df = 48035, p-value < 2.2e-16
## alternative hypothesis: true correlation is not equal to 0
## 95 percent confidence interval:
## -0.7223805 -0.7137172
## sample estimates:
## cor
## -0.7180766
